# Analysis of cattle movement networks in Paraguay: Implications for the spread and control of infectious diseases

**DOI:** 10.1371/journal.pone.0278999

**Published:** 2022-12-19

**Authors:** Amaias Avalos, Benoit Durand, José Naranjo, Victor Maldonado, Laetitia Canini, Gina Zanella

**Affiliations:** 1 ANSES, Laboratory for Animal Health, Epidemiology Unit, Paris-Est University, Maisons-Alfort, France; 2 Faculté de Médecine, Université Paris-Saclay, Le Kremlin-Bicêtre, France; 3 National Animal Health and Quality Service (SENACSA) Consultant—Animal Health Services Foundation (FUNDASSA), Mariano Roque Alonso, Paraguay; 4 National Animal Health and Quality Service (SENACSA), General Directorate of Animal Health, Identity and Traceability, San Lorenzo, Paraguay; Swedish National Veterinary Institute, SWEDEN

## Abstract

Beef exports represent a substantial part of Paraguay’s agricultural sector. Cattle movements involve a high risk due to the possible spread of bovine diseases that can have a significant impact on the country’s economy. We analyzed cattle movements from 2014 to 2018 using the networks analysis methodology at the holding and district levels at different temporal scales. We built two types of networks to identify network characteristics that may contribute to the spread of two diseases with different epidemiological characteristics: i) a network including all cattle movements to consider the transmission of a disease of rapid spread like foot and mouth disease, and ii) a network including only cow movements to account for bovine brucellosis, a disease of slow spread that occurs mainly in adult females. Network indicators did not vary substantially among the cattle and cow only networks. The holdings/districts included in the largest strongly connected components were distributed throughout the country. Percolation analysis performed at the holding level showed that a large number of holdings should be removed to make the largest strongly connected component disappear. Higher values of the centrality indicators were found for markets than for farms, indicating that they may play an important role in the spread of an infectious disease. At the holding level (but not at the district level), the networks exhibited characteristics of small-world networks. This property may facilitate the spread of foot and mouth disease in case of re-emergence, or of bovine brucellosis in the country through cattle movements. They should be taken into account when implementing surveillance or control measures for these diseases.

## Introduction

Animal trade is an important mode of transmission of infectious diseases [1], such as foot and mouth disease (FMD) and brucellosis, two major diseases of livestock [2–7].

FMD is caused by a virus of the genus *Aphthovirus*, family *Picornaviridae*. There are seven serotypes of FMD virus, namely O, A, C, SAT 1, SAT 2, SAT 3 and Asia 1, which infect cloven-hoofed animals. Infection with one serotype does not confer immunity against another [8–10]. Ruminants of both sexes and of all age categories may be infected and their trade may allow transmission of the virus from farm to farm. For this reason, FMD is considered a transboundary animal disease. It is an economically important disease due to direct losses for farmers [11] and trade barriers [12], which justifies the implementation of surveillance and control programs. In South America, the Pan American Foot and Mouth Disease Center (PANAFTOSA), within the Pan American Health Organization (PAHO), has supervised these programs since their creation in 1951, in agreement with the Organization of American States (OAS) and the Government of Brazil, [7, 13, 14]. In 1988, PANAFTOSA proposed a preliminary action plan for the eradication of FMD, the implementation of which allowed most South American countries to obtain and consolidate a status of FMD-free countries where vaccination is not practiced (Chile, Peru) or practiced (Paraguay and Uruguay), or where vaccination is practiced alongside non-vaccination zones (Argentina, Bolivia, Brazil, Colombia and Ecuador) [15]. The 2021–2025 action plan aims to eliminate the use of vaccination in order to achieve the FMD-free status without vaccination, thereby gaining access to a more economically profitable market than with vaccination.

Brucellosis is a zoonotic disease, considered extremely infectious by the OIE, and is transmitted by contact between infected animals, and from infected animals to humans [16]. The disease is caused by several bacteria belonging to the genus *Brucella*, which affects many mammalian species, including cattle, goats, pigs, and sheep [3, 17]. Bovine brucellosis (BB), predominantly caused by *Brucella abortus*, mainly affects pregnant females that abort, and trade of heifers and cows may allow transmission of the bacteria from farm to farm. Human exposure is occupational (i.e., in livestock farmers, veterinarians and slaughterhouse personnel), but is also linked to consumption of infected milk and milk products [18–20]. Control of BB in animal populations is therefore important to improve the productive capacity of the herd and to protect human health from future infections [21]. Brucellosis is present in South America [22, 23] and has been reported in Argentina [24, 25], Brazil [26] and Paraguay [27], among others [28]. Eradication by testing and culling is a costly investment for the producer without government assistance, which is the case in the vast majority of South American countries, where the producer absorbs most of the control measure costs. Vaccination with S19 or RB51 vaccines is an effective method of preventing and controlling infection, but it requires strict control of animal movements and the application of rigorous rules when introducing breeding animals into the herd [24, 26].

Paraguay is a country with a large beef cattle production sector and has great potential to be among the world’s largest beef exporters [27]. FMD and BB are the two animal diseases that the Paraguayan health authorities consider priorities for control and eradication. Since its creation in 1967, the National Animal Health and Quality Service (SENACSA) has worked for animal health in Paraguay. At that time, the main objective was to implement an FMD control program, which began in 1968 and was a component of regional level disease control activities. In 1992, an official FMD Eradication Program was initiated with the implementation of vaccination, surveillance, and stamping-out of part of the FMD outbreaks, to eventually allow farmers access to the international commercial market for animals and meat [6, 29]. The last reported outbreak of FMD in Paraguay occurred in 2012 and led to increasingly strict and severe surveillance and control measures until recovery of the “Free of FMD with vaccination” status in 2017 that has been maintained until now [30, 31].

Currently, epidemiological surveillance of BB in Paraguay consists of the collection of information from control activities carried out by official veterinarians, which is processed by local, regional, and central health offices. Official veterinarians perform serological control tests with a testing protocol (Rose Bengal test and fluorescence polarization assay) on females and males introduced on farms that are willing to obtain an official certificate as free from brucellosis, as well as milk ring-tests on certain dairy farms. The current BB control, prevention, and eradication program driven mainly by SENACSA does not include, in the short term, the elimination of positive animals with compensation to owners, as established by the Law for the Promotion of Dairy Production (Law 5264/2014), mainly due to lack of funds. Two vaccination campaigns should be carried out per year on all farms in young females from 3 to 8 months of age, using the S19 and RB51 strains.

It is well known that animal movements play an important role in disease transmission between farms. Data on the commercial movements of animals can be represented by means of networks [4, 32–34]. The properties of these networks can be studied using network analysis methods [35].

The main route of BB transmission between farms is the trade of infected cows. For FMD, other routes than cattle trade may play a role in the transmission of the disease between farms: local airborne contagion, pig and small ruminant movements or movements of people, vehicles and livestock equipment. Creating national databases is an important requirement for the control of diseases such as FMD [36]. In Paraguay, the main domestic species raised is cattle (96% of the livestock that includes cattle, sheep, goats and pigs [37] and, therefore, cattle trade could be considered as the major risk for FMD propagation. In 2008, the cattle movement information system, called SIGOR, was developed by SENACSA in the frame of the FMD control campaign [29]. Considering that cattle trade is the main source of BB and FMD spread in Paraguay and that network analysis may enable better understanding of the propagation of an exotic disease (such as FMD in Paraguay), in the case of its introduction, or the mechanisms of circulation of enzootic diseases (such as BB in Paraguay) [38–42], the objective of this work was to analyze and compare two cattle trade networks in Paraguay: the general cattle trade network (relevant for FMD), and the cow trade network (relevant for BB). Both networks were studied during the period 2014–2018 and analyzed at holding and district levels, for the entire period or using monthly and annual time steps.

## Materials and methods

### Data

Information on the cattle inventory, list of cattle markets and slaughterhouses, and cattle movements from 2014 to 2018 was obtained from the Paraguayan Veterinary Services (SENACSA).

The database on cattle inventory gathered information on farm location, specifying three administrative units (from the smallest to the largest: district, department and region) and the number of cattle per farm. This information was used to establish two maps of farm and cattle densities per km^2^ using the average of annual numbers of farms and cattle from 2014 to 2018.

Data on cattle movements from January 2014 to December 2018 were extracted from the cattle movement database that was developed in the context of the Paraguayan national control program for FMD. Each movement included the following information: date, holding identification number, type of holding at origin and destination (farm, market, or slaughterhouse), location, and number of animals moved by category (cows, heifers, bullocks, bulls, steers, weaned male/female and calves). Movements concerning import or export were excluded.

### Network construction and analysis

We aggregated the movement data to construct static networks in which the nodes were either holdings (farms or markets, referred to below as “holding level”) or districts (referred to below as “district level”) and cattle movements between nodes were links. The networks were directed because the origin and destination of cattle movements were taken into account. Slaughterhouses were not included in the analysis since they do not play a role in the spread of pathogens. Considering that FMD or BB propagation depend on the cattle categories they may affect, we differentiated networks including all cattle movements (referred to below as “all cattle networks”) from networks including only cow movements (referred to below as “cow networks”). Time interval was either the entire period (“global network”), one of the 5 years (“annual network”), or one of the 60 months (“monthly networks”) in our data set. The global or annual scales would be more suitable for diseases of slow spread that are enzootic, such as brucellosis, to identify patterns that could assist in the implementation of control measures. For a disease that spreads faster, such as foot and mouth disease, the monthly networks would be more adapted. A month would be the period of time in which animals in a herd are still infectious and could propagate the infection through movements. Indeed, it has been reported that in a herd of vaccinated cattle, FMD clinical signs may be displayed over a 39-day period [43] and, by modelling, it has been found that transmission within a cattle farm can occur until one month post-infection [44]. All scales were studied for both all cattle and only cow networks to compare them. The different combinations yielded a total of 264 networks. Different indicators were calculated for all networks and are briefly described in **Table 1**.

**Table 1:** Description of general network indicators calculated in this study. *Calculated for the global and annual networks.

Network indicator	Description
**Size**	Number of nodes [35]
**Density**	Number of existing links divided by the total possible links [35]
**Diameter**	The most extensive shortest path among all the shortest paths in the network [45]
**Average degree**	The degree of a node is the number of links it has with other nodes [35]
**Betweenness**	Frequency with which a node appears on the shortest path between other pairs of nodes [46]
**Average path length**	Average number of links along the shortest paths for all possible pairs of nodes [35]
**Assortativity**	Tendency of nodes to have links with similar nodes in terms of degree [47]
**Clustering coefficient**	Proportion of nearby nodes of a node that are linked to each other [48]
**Reciprocity**	Proportion of nodes in a directed network that are mutually linked [35]
Connected component*	Sub-network where all nodes are connected. In a strongly connected component, any node can be accessed by any other node through directed links, while in a weakly connected component, all nodes are linked without taking into account the direction of the links [49, 50]
Closeness*	Inverse of the average length of the shortest paths to/from all other nodes in the graph (the largest facilities will have high and fast accessibility to anywhere in the network). It was calculated within the largest strongly connected component [46]
Community*	Densely connected subgraphs identified using an algorithm based on random walks, the walktrap algorithm [51]

We calculated the Jaccard coefficient for the different pairs of networks to compare the nodes and links included in each network, from year to year and from month to month. The Jaccard index measures the degree of similarity between two sets A and B (regardless of the type of elements) with the formula:

J(A,B)=|A∩B|/|A∪B|

It has a value between 0 (no similarity) and 1 (identical sets).

To establish whether the networks had scale-free properties, whose degree distribution follows a power law, we plotted the degrees on a logarithmic scale [52]. When relevant, the exponent of the power law distribution was determined using the method proposed by Clauset *et al* [53]. We also generated random networks according to the Erdos-Rényi model [54], with the same number of nodes and links as real networks and compared their average path length and clustering coefficient to those of the real networks to detect scale-free or small world properties [35, 55].

Finally, percolation allowed us to analyze the effect of eliminating nodes on the size of the largest strongly connected component (LSCC), to assess control measures that could be implemented to prevent the spread of bovine infectious diseases. The percolation analysis was performed at the holding level for the global and annual networks for all cattle and cows. Considering the results obtained in other studies [39, 42], nodes with high betweenness were sequentially removed to identify a threshold at which the LSCC would rapidly reduce in size.

Network analyses were performed using the Igraph Network Analysis and Visualization package [56], ggplot packages [57], and RStudio software (v 1.4.1106, 2009–2021) [58].

## Results

### Cattle census description

According to data from the 2014–2018 cattle census, there were an average of ~14 million cattle and ~148,000 holdings in Paraguay during the study period **(Table 2)**.

**Table 2:** Number of cattle and cattle farms censused in Paraguay from 2014 to 2018.

	2014	2015	2016	2017	2018
**Number of cattle farms**	147,320	151,084	150,689	148,536	145,025
**Number of cattle**	14,465,581	14,216,256	13,858,584	13,821,526	13,500,965

**Fig 1:**
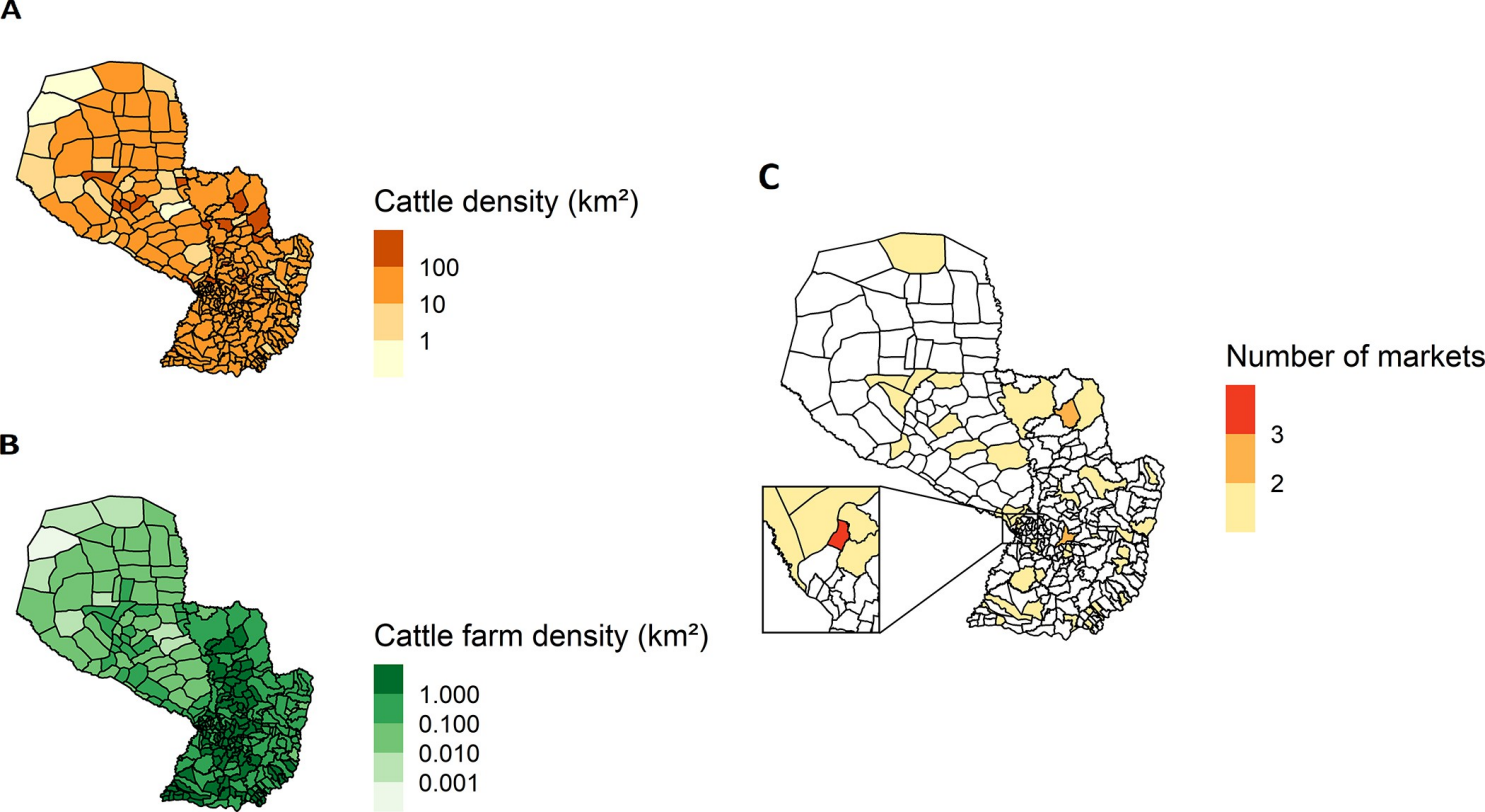
Density of cattle farms and cattle per km^2^ and market location in Paraguay. Average annual number of farms and cattle from 2014 to 2018 were used.

The average cattle density was high throughout the country. Cattle farms were present all over the Paraguayan territory, with a higher density in the eastern region compared to the western region called El Chaco **(Fig 1)**. A total of 54 markets and 312 cattle slaughterhouses were active during the study period **(Fig 1)**. The highest number of markets was concentrated in the eastern region of Paraguay.

### Cattle movement database description

According to the database, a total number of 73,904 farms were involved in cattle movements during the study period, representing ~50% of the total number of farms present in the cattle census conducted by SENACSA. The Chaco region had 402,289 origin movements and 318,055 destination movements; while the eastern region had a higher number of origin movements (1,000,121) and destination movements (1,084,355).

The highest number of movements between 2014 and 2018 occurred from farm to farm, regardless of the categories of animals moved (685,591 for all cattle and 194,388 for only cows), followed by movements from farms to slaughterhouses (416,738 for all cattle and 178,412 for only cows) **(Table 3)**. The main destination of movements originating at markets were the slaughterhouses. The number of cow movements was 37% of all cattle movements (523,597 of 1,402,410). The number of cows moved from farms to slaughterhouses (3,050,147) exceeded that from farms to farms (2,340,498), while for the non-cows, the animals moved from farms to slaughterhouses (7,352,360) were half of those moved from farms to farms (12,061,614).

**Table 3:** Summary of cattle movements within and between districts in Paraguay from 2014 to 2018. *The percentages represent the proportion of movements that occurred within districts.

	Destination	Number of movements *(% within district)				Number of moved cattle *(% within district)			
	Origin	Farm	Market	Slaughterhouse	Total	Farm	Market	Slaughterhouse	Total
**All cattle**	Farm	685,591 (40%)	68,691 (5%)	416,738 (16%)	**1,171,020 (30%)**	15,319,772 (29%)	1,381,055 (6%)	11,353,034 (6%)	**28,053,861 (18%)**
	Market	68,799 (11%)	34 (6%)	162,557 (28%)	**231,390 (23%)**	670,144 (11%)	265 (2%)	661,144 (31%)	**1,331,553 (21%)**
	**Total**	**754,390 (38%)**	**68,725 (5%)**	**579,295 (19%)**	**1,402,410 (28%)**	**15,989,916 (28%)**	**1,381,320 (6%)**	**12,014,178 (7%)**	**29,385,414 (19%)**
**Only cows**	Farm	194,388 (50%)	39,706 (3%)	178,421 (22%)	**412,515 (33%)**	2,340,498 (39%)	533,432 (2%)	3,050,147 (9%)	**5,924,077 (20%)**
	Market	21,615 (11%)	13 (0%)	89,454 (28%)	**111,082 (25%)**	159,974 (10%)	43 (0%)	350,233 (28%)	**510,250 (23%)**
	**Total**	**216,003 (46%)**	**39,719 (3%)**	**267,875 (24%)**	**523,597 (32%)**	**2,500,472 (11%)**	**533,475 (2%)**	**3,400,380 (37%)**	**6,434,327 (20%)**
Excluding cows*	Farm	491,203 (36%)	28,985 (8%)	238,317 (28%)	**758,505 (25%)**	12,061,614 (25%)	667,583 (10%)	7,352,360 (5%)	**20,081,557 (17%)**
	Market	47,184 (11%)	21 (10%)	73,103 (21%)	**120,308 (32%)**	489,634 (11%)	185 (2%)	293,698 (33%)	**783,517 (20%)**
	**Total**	**538,387 (34%)**	**29,006 (8%)**	**311,420 (27%)**	**878,813 (25%)**	**12,551,248 (24%)**	**667,768 (10%)**	**7,646,058 (6%)**	**20,865,074 (17%)**

*Males, calves and heifers

Most cattle movements occurred between districts (72%). This percentage dropped to 60% when movements occurred only between farms, and to 50% when only cows were taken into account.

### Size and stability of networks

#### Holding level

The global network was composed of 72,096 nodes and 366,626 links for all cattle, and 57,097 nodes and 126,359 links for only cows **(Table 4)**.

**Table 4:** Indicators for the global networks for all cattle and cows in Paraguay. Nodes and links were aggregated from 2014 to 2018.

	Holding level		District level	
	All cattle (range)	Cows (range)	All cattle (range)	Cows (range)
**Number of nodes**	72,096	57,097	299	299
**Number of links**	366,626	126,359	22,077	11,306
**Diameter**	20	26	4	4
**Density**	7.05.10^−05^	3.87.10^−05^	0.25	0.12
**Average degree**	10.17 (1–4,669)	4.42 (1–3,261)	147.67 (2–466)	75.62 (2–411)
**Average in-degree**	5.08 (0–3,948)	2.21 (0–2,878)	73.83 (1–235)	37.81 (1–228)
**Average out-degree**	5.08 (0–1,215)	2.21 (0–392)	73.83 (1–231)	37.81 (1–183)
**Average betweenness**	7.89.10^−10^ (0–2.72.10^−06^)	7.74^−10^ (0–2.82.10^−06^)	1.78.10^−05^ (0–0.0003)	2.32.10^−05^ (0–0.0007)
**Average path length**	5.53	7.18	1.79	2.03
**Assortativity**	-0.04	-0.03	-0.06	-0.06
**Clustering coefficient**	0.02	0.01	0.57	0.39
**Reciprocity**	0.05	0.04	0.38	0.34
**Strong components**				
Number	39,639	43,727	1	1
Largest component size (% total nodes)	32,149 (44%)	12,390 (21%)	299 (100%)	299 (100%)
**Weak components**				
Number	913	2,777	1	1
Largest component size (total nodes)	70,070 (97%)	50,593 (88%)	299 (100%)	299 (100%)
**Average closeness**	0.19 (0.09–0.30)	0.15 (0.07–0.21)	0.56 (0.38–0.81)	0.49 (0.32–0.72)
**Communities**				
Number	3,589	6,264	5	8
Size of the first three largest communities (% total nodes)	16,288 (40%) - 7,076–5,311	11,630 (38%) - 5,992–4,139	120 (92%) - 100–56	77 (63%) - 61–53

**Fig 2:**
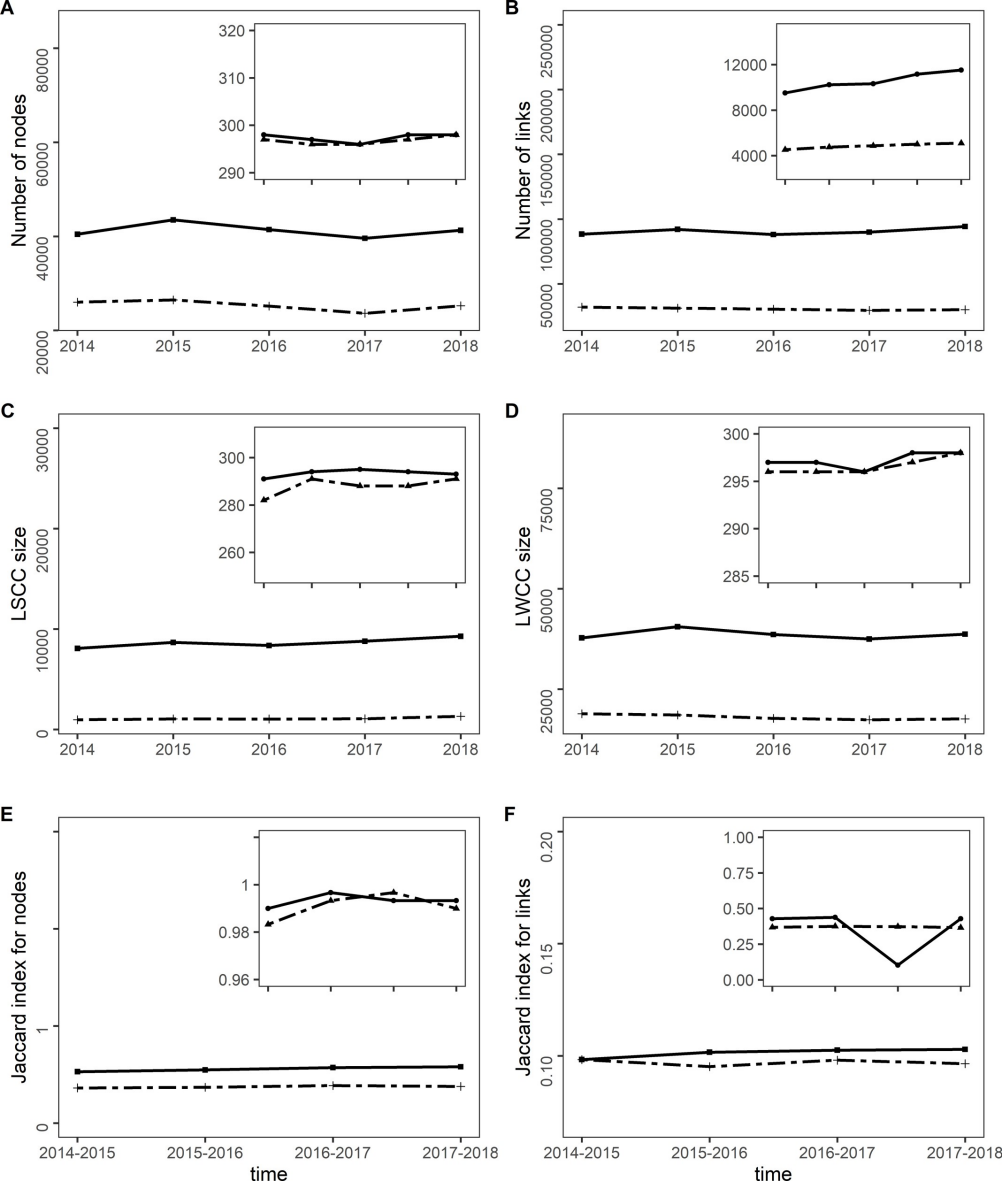
Nodes, links, largest connected components sizes and Jaccard index for the annual networks. (A) Number of nodes, (B) Number of links, (C) Largest strongly connected component (LSCC) size, (D) Largest weakly connected component (LWCC) size, (E) Jaccard index for nodes, and (F) Jaccard index for links at the holding (main graphs) and district levels (insets) for all cattle (plain lines) and for only cows (dashed lines).

In the annual networks, the average numbers of nodes and links for all cattle were 41,251 (range: 39,589–43,496) and 90,556 (range: 88,101–94,295), respectively, of which 61.3% of nodes and 33.9% of links corresponded to only cows **(Fig 2)**. The Jaccard index (JI) values for the nodes of the two types of annual networks (all cattle and cows) were around 0.5, indicating that almost 50% of the same holdings were involved in cattle trade from one year to the next **(Fig 2)**. Forty-eight percent of the nodes of the all cattle global network were present in one or two years and 25% in all the five years. The JI values for the links ranged from 0.1 for all cattle to values <0.1 for only cows, which means that a small part of the exchanges were made between the same farms from one year to the next. Sixty percent of the nodes of the cow global network were present in one or two years and 10% in all the five years.

For the monthly networks, there was an average of 9,436 nodes, (range: 2,035–15,499) and an average of 9,283 links (range: 1,919–16,240) for all cattle **(S1 Fig)**. Very close figures were obtained for the only cow monthly networks. The monthly values of the JI **(S2 Fig)** at the holdings level for all cattle and only cows only ranged from 0.6 to 1 for nodes and from 0.15 to 0.35 for links. Fifty-three percent of the nodes were present in 4 or less months for all cattle and only cow networks.

#### District level

The global network included 299 nodes for both all cattle and cow networks. This means that all Paraguayan districts presented in this study were involved in cattle trade. The number of links of the global cow network represented 50% of the number of links of the global network for all cattle **(Table 4)**.

The number of nodes in the annual networks was high and close to the number of nodes in the global network. The number of links in the cow networks was half that in the all cattle networks **(Fig 2)**. At the district level, the JI values of nodes were close to 1 for the all cattle and cow networks; these values were higher compared to those for holdings **(Fig 2)**. The JI values for the links ranged between 0.1 and 0.4 for all cattle; 0.36 and 0.37 for only cows.

In the monthly networks, there was an average of 282 nodes (range: 240–291) and an average of 2,443 links (range: 936–3,500) for all cattle **(S1 Fig)**. For only cows, the means of nodes and links represented 92% and 43%, respectively, of the mean total number of nodes and links. For the holding level, the mean JI for nodes was 0.18 (range: 0.08–0.27) for all cattle, and 0.11 (range: 0.05–0.15) for only cows **(S2 Fig)**. Mean JI values for links were 0.03 (range: 0.01–0.04) for all cattle, and 0.03 (range: 0.01–0.05) for only cows.

### Connected components analysis

#### Holding level

**Fig 3:**
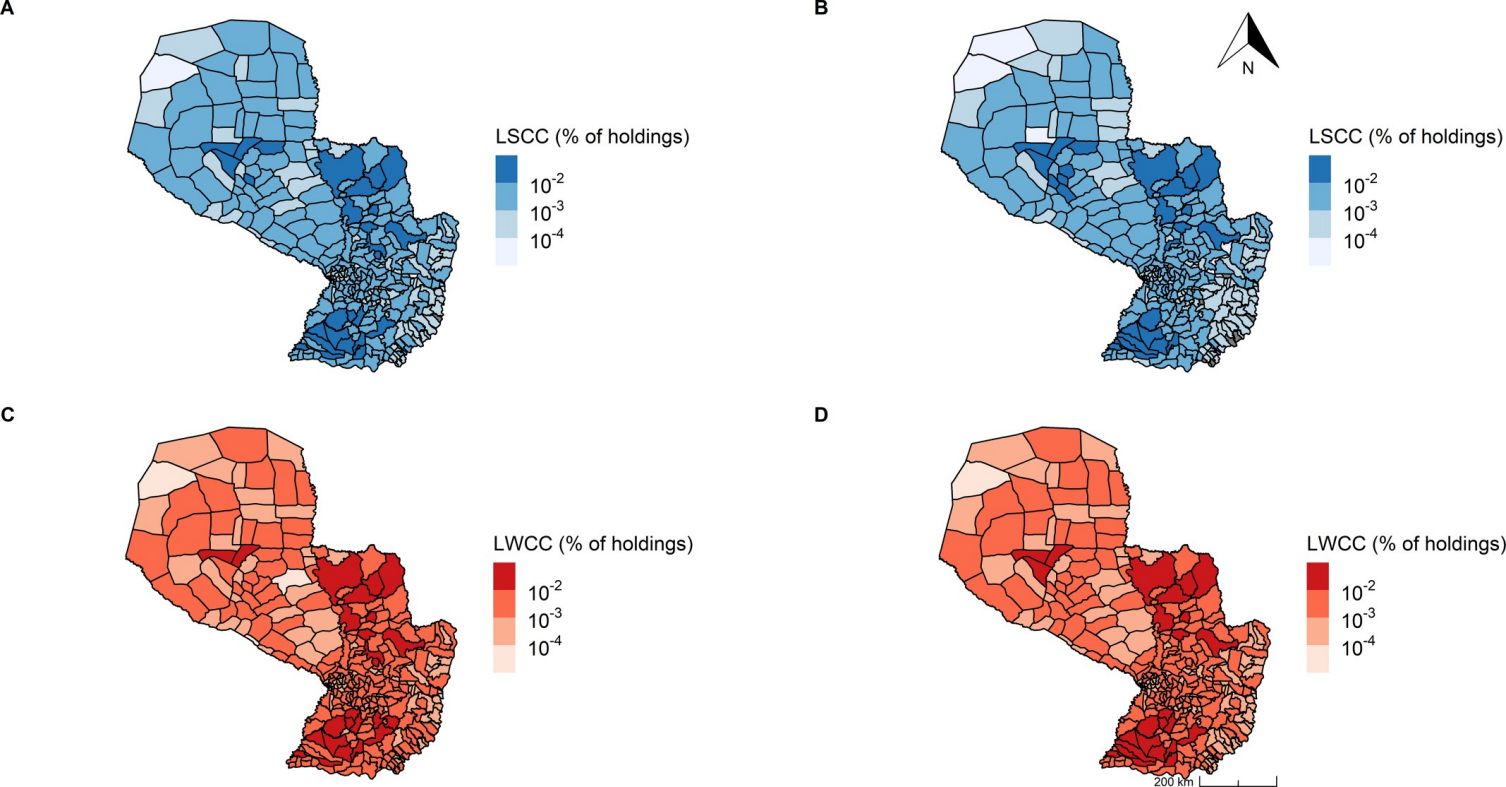
Spatial distribution of the holdings in the largest strongly and weakly connected components. Largest strongly connected component (LSCC) (A): all cattle, (B): cows and largest weakly connected component (LWCC) (C): all cattle, (D): cows for the global network (aggregated nodes and links from 2014 to 2018). Percentage of holdings in each district corresponds to the number of holdings belonging to the largest component over the size of that largest component.

The number of nodes included in the largest strongly connected component (LSCC) represented 44% of the nodes of the global network for all cattle and 21% for cows **(Table 4)**. Holdings included in both LSCCs were distributed all over the country **(Fig 3)**.

In the case of annual networks, the average size of the LSCCs in holdings was 8,635 for all cattle (range: 8,073–9,282) and 1,097 for cows (range: 980–1,319), which represents approximately 21% and 4.3% of the total number of nodes for all cattle and cows **(Fig 2)**. For the monthly networks, the average size of the LSCCs was 139 for all cattle (range: 18–326) and 30 for cows (range: 5–77); this average represented 1.5% of the average total number of nodes for all cattle and 0.8% of the average total number of nodes for cows. The LSCCs size showed a seasonality mainly for the all cattle networks: it was higher from July to October (**S3 Fig**).

The largest weakly connected component (LWCC) included almost all nodes in the global network for all cattle (97%) and (88%) for cows **(Table 4, Fig 3).** For the annual networks, the LWCC average size was 38,632 nodes for all cattle (range: 37,496–40,561) and 18,054 nodes for cows (range: 17,413–18,893), representing 93% and 71% of the average total number of nodes for all cattle and cows, respectively. For the monthly networks, the LWCC had a mean of 5,688 nodes for all cattle (range: 906–10,810) and 1,084 nodes for cows (range: 354–2,239), representing 58% and 27% of the average total number of nodes for all cattle and cows.

#### District level

All districts were included in the global LSCC and LWCC for all cattle and cow networks **(Table 4)**. In the annual networks, around 97% of all districts were included in the LSCC for all cattle and only cow networks. For the monthly LSCC network, an average of 87% of the total number of nodes was observed for all cattle networks, and 65% for only cow networks. Almost all districts were included in the LWCC of all the annual and monthly networks.

### Communities (global networks)

**Fig 4:**
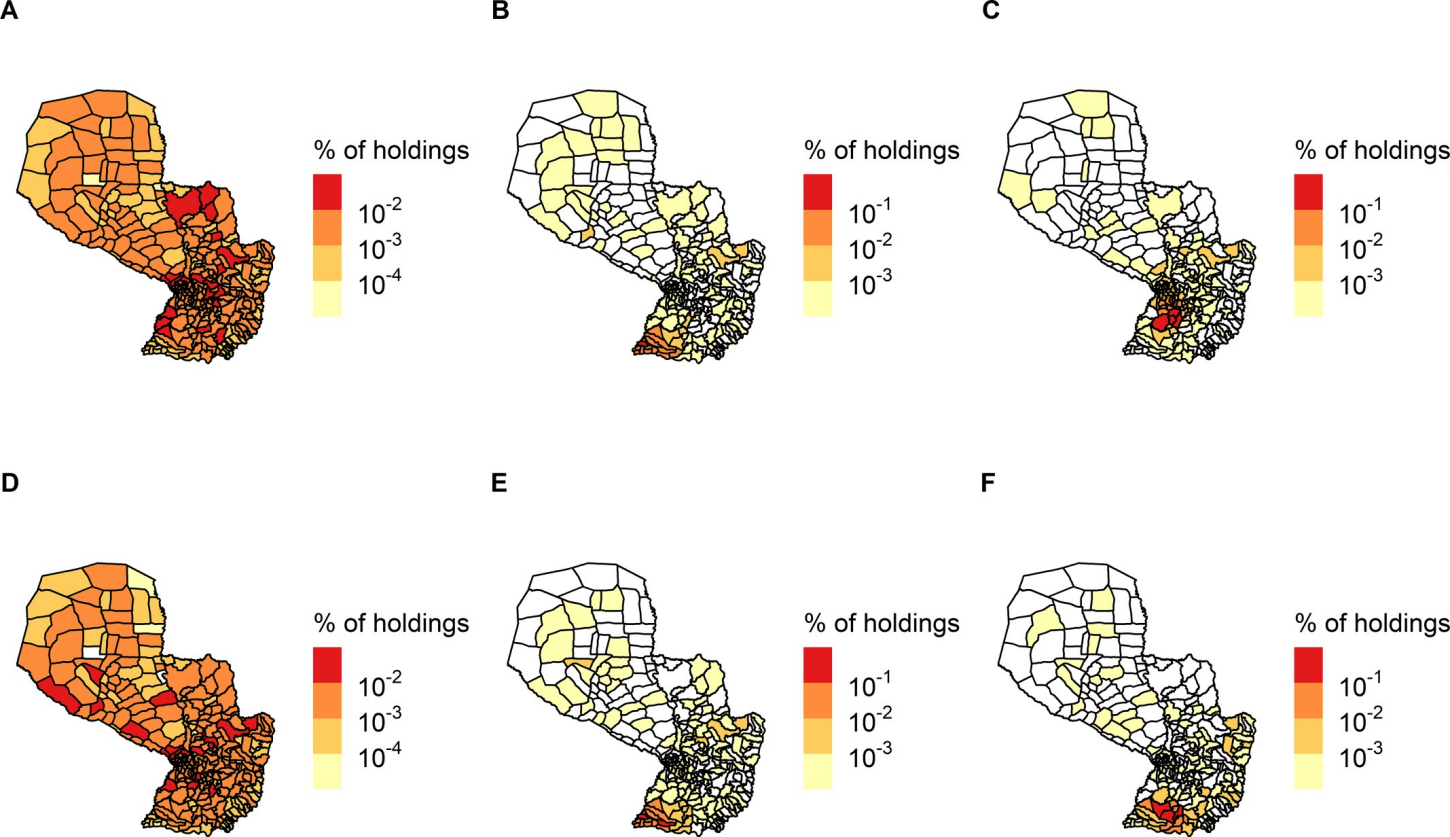
Spatial distribution of the first three communities of the global networks at the holding level. Communities (A-B-C): all cattle and (D-E-F): only cows in Paraguay (aggregated nodes and links from 2014 to 2018). Percentage of holdings in each district corresponds to the number of holdings belonging to the community over the size of that community.

At the holding level, the first three communities represented 40% of the total number of nodes for the all cattle global network and 38% for the cow global network **(Table 4, Fig 4)**. The spatial distribution of the holdings included in the first community for both global networks covered most of the country.

**Fig 5:**
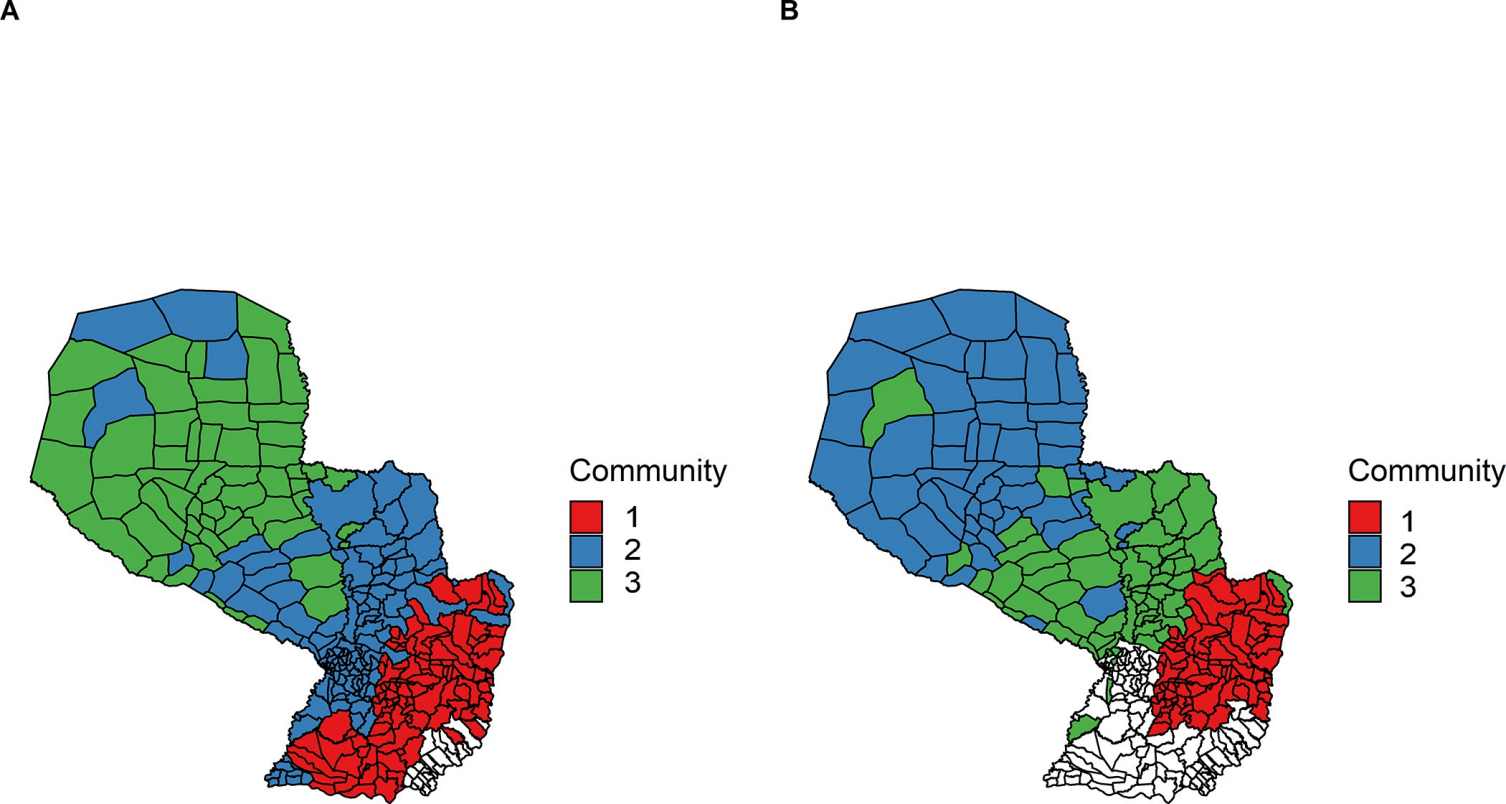
Spatial distribution of the first three communities of the global networks at the district level. (A) All cattle and (B) only cows. Nodes and links were aggregated from 2014 to 2018.

At the district level, there were fewer communities **(Table 4)** and the districts belonging to the first three communities were more geographically clustered **(Fig 5)**, representing 92% of the total number of nodes for the all cattle global network and 63% for the only cow global network.

### Network indicators

#### Centrality indicators

**Fig 6:**
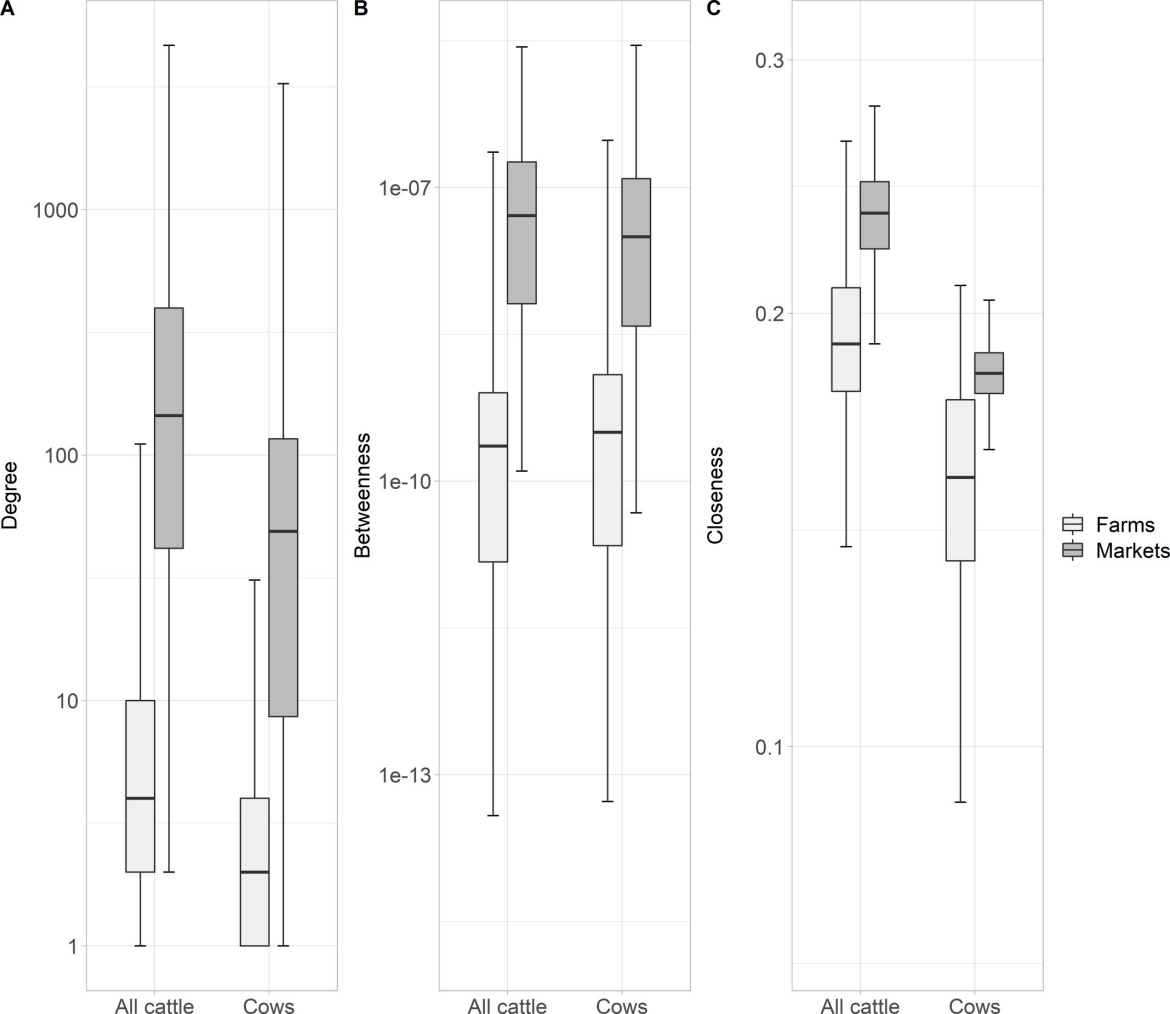
Distribution of centrality indicators for all cattle and cows in global networks differentiating farms and markets. (A) Degrees, (B) betweenness, and (C) closeness calculated in the largest strongly component (nodes and links aggregated from 2014 to 2018).

**Fig 7:**
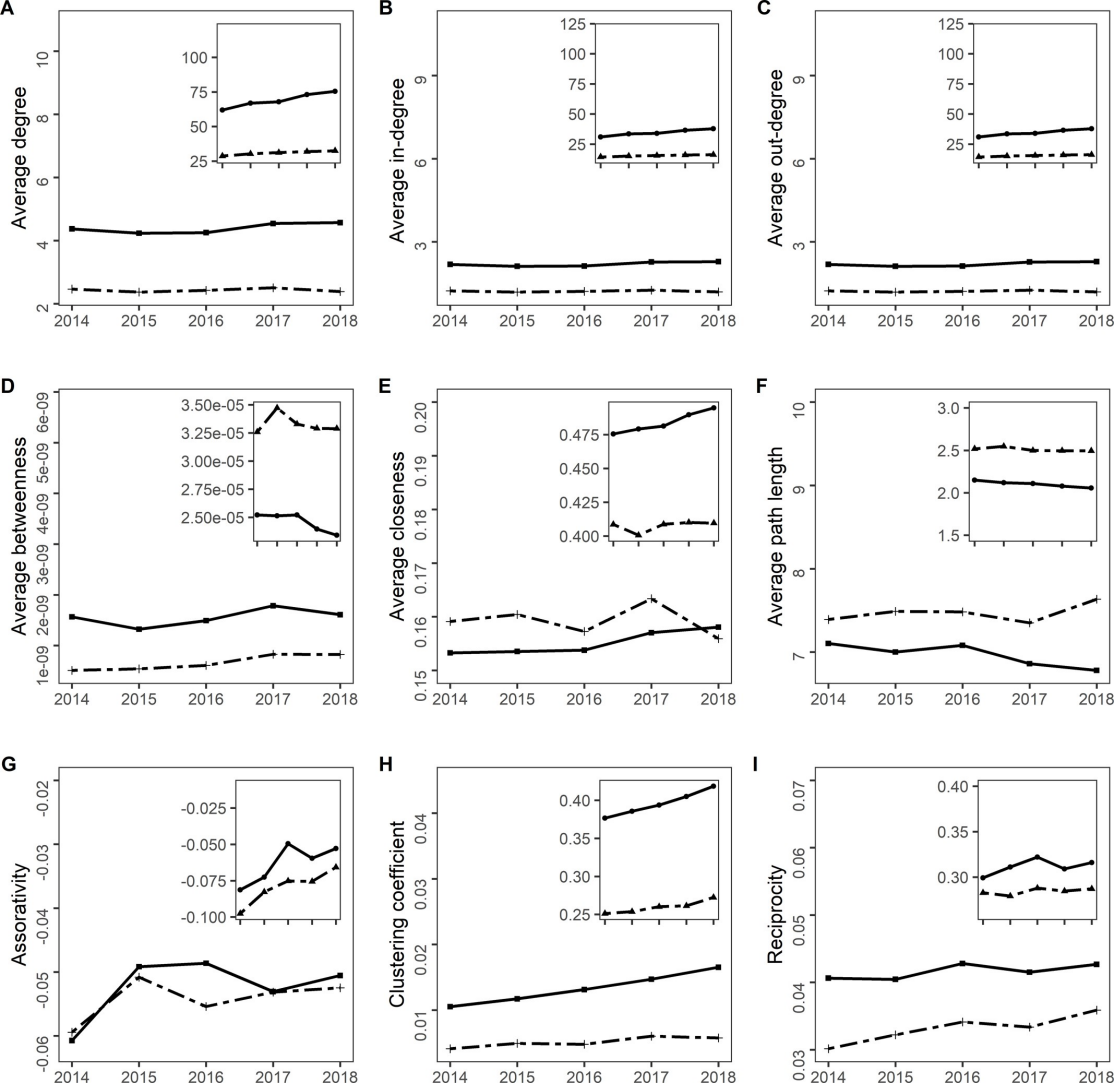
Network indicators for the annual networks for all cattle and only cows in Paraguay. Holding level (main graphs), district level (insets), all cattle (plain line) and only cows (dashed line).

At the holding level, the node degrees in the global network for all cattle ranged from 1 to 4,669, with an average value of 10.17 (**Table 4**). This average value was lower for the global cow network (4.42), with degrees ranging from 1 to 3,260. Average in-degree and out-degree values had the same the same values for the global networks, however, the maximum value was higher for the in-degree. Average betweenness and closeness were low and similar for both networks. However, when distinguishing the nodes by type of holding (farms *vs* markets), significantly higher values of the three centrality indicators were found for markets for all cattle (Wilcoxon’s tests, *p*<0.0001) **(Fig 6)**. Centrality indicators for the annual networks were similar to those for the global networks **(Fig 7)**. The average of the node degrees for the five years ranged from 4.23 to 4.56 for all cattle, and from 2.36 to 2.50 for only cows; the average in-degree was of 2.12 for all cattle and 1.18 for only cows for each year; the out-degree value was the same for each year (2.284 for all cattle and 1.25 for only cows); the average betweenness 1.32.10^−9^ and 1.78.10^−9^ for all cattle, and 5.10^−10^ and 8.29.10^−10^ for only cows; and the average closeness 0.15 and 0.16 for all cattle, and 0.15 and 0.16 for only cows. For the monthly networks, the average degree varied between 1.70 and 2.10 for all cattle; in cow networks the values were lower. Concerning betweenness, the values ranged from 3.61.10^−7^ to 2.48.10^−5^ for all cattle, and from 3.92.10^−7^ to 4.88.10^−5^ for only cows **(S1 Fig)**.

At the district level, the node degrees in the global network for all cattle presented values between 2 and 466, with a mean value of 147 **(Table 4)**; the values for only cow networks were lower. Average values for betweenness for all cattle were 1.78.10^−5^ (0–3.10^−4^) and were higher for the only cow networks. Closeness ranged from 0.38 to 0.81 for all cattle, and from 0.32 to 0.72 for the only cow networks. For the annual networks, the average degree ranged from 63.83 to 77.26 for all cattle, and from 30.48 to 34.20 for only cow; the betweenness 2.34.10^−5^ to 2.52.10^−5^ for all cattle, and 3.25.10^−5^ to 3.47.10^−5^ for only cows; and the closeness around 0.47 to 0.49 for all cattle and 0.40 to 0.41 for only cows. For monthly networks, the average degree presented values between 7.7 and 24, with lower values in cows (4.5–10.7), for betweenness values ranged from 0.005 to 0.008 for all cattle, and 0.003 to 0.009 for only cows.

#### Other indicators

Density values were slightly higher for the all cattle global networks compared to those for the only cow networks at the holding level **(Table 4)**. The holdings in global networks were linked on average by fewer edges in the all cattle global network (average path length: 5.53) than in the only cow global network (average path length: 7.18) **(Table 4)**. The diameter at the holding level was 20 for all cattle and 26 for only cows **(Table 4)**. Assortativity was negative for all the networks at the holding level, indicating that nodes were more frequently linked to nodes with a different degree. Clustering coefficient values were lower for the networks at the holding level. The low reciprocity values obtained for all networks, mainly at the holding level, indicated that very few holdings received cattle from holdings to which they sent animals. **(Fig 7)**. For the annual networks, densities ranged from 4.86.10^−05^ to 5.73.10^−05^ for all cattle and 4.47.10^−05^ to 5.29.10^−05^ for only cows. There were also on average the same number of steps as in the global networks for all cattle (average path length: 6.7–7.1) and only cows between 7.3 and 7.6. The diameter ranged from 24 to 29 for all cattle, and from 24 to 31 for only cows. The monthly networks had densities between 6.76.10^−5^ and 4.63.10^−4^ for all cattle and for only cows, the average path length at the holding level ranged from 2.5 to 8.2 for all cattle, and 2 to 6 for only cows, the diameter ranged from 7 to 25 for all cattle and for only cows **(S1 Fig)**.

As for the district level, the density value was higher for the all cattle global network **(Table 4)**. Average path length values followed the same trend, as well as the average path length values at the holding level **(Table 4)**. The diameter was 4 for both all cattle and only cow networks **(Table 4)**. For the annual networks, densities ranged from 0.107 to 0.130 for all cattle, and from 0.051 to 0.057 for only cows, and the diameter from 4 to 5 for all cattle, and 4 to 6 for only cows. For both the all cattle and only cows networks, the monthly network densities ranged from 0.01 to 0.04, and the diameters from 6 to 14.

### Global network topology

#### Holding level

**Fig 8:**
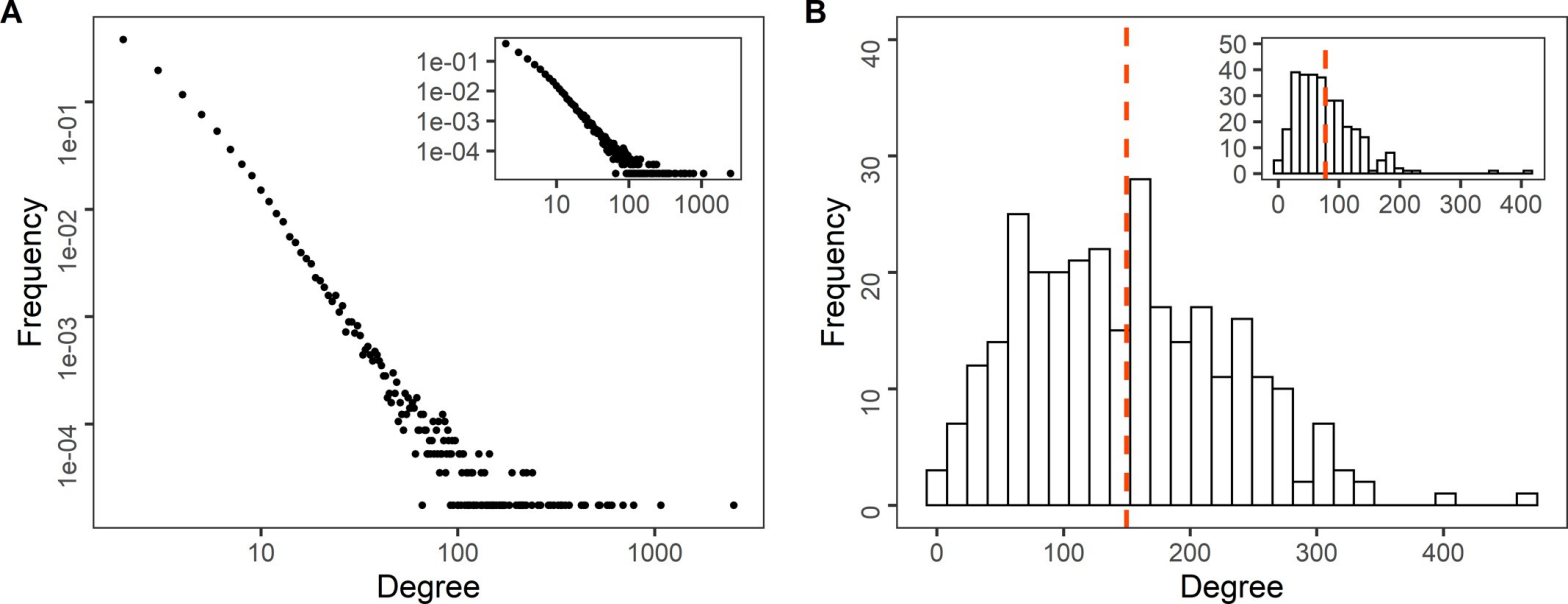
Degree distribution in a logarithmic scale for all cattle and cows in the global networks. (A) Holding level, (B) district level, all cattle (main graph) and cows (inset) (nodes and links were aggregated from 2014 to 2018).

The degree distribution in a log-log scale for both the all cattle and only cow global networks showed a linear shape **(Fig 8)** and appeared heavy tailed, suggesting a scale-free structure for both types of network. However, there were only two orders of magnitude between the minimum degree and maximum degree, which do not allow to conclude about a scale-free property of the networks. Nevertheless, we calculated the variance to mean degree ratio, which is linked to the basic reproductive rate *R*_*o*_ for an infection transmitted across a network [59]. The ratio was 127 for the all cattle network and 106 for the only cow network, indicating a strong heterogeneity between nodes. The clustering coefficient values of the real networks were much higher than those of the random networks **(Table 5)**, while the values of the average path length were close, indicating that they exhibited small-world properties.

**Table 5:** Clustering coefficient and average path length for the global real and random networks in Paraguay. The nodes and links were aggregated from 2014 to 2018.

		Indicators	Real	Random
**Holding level**	All Cattle	Clustering coefficient	0.023	0.00015
		Average path length	5.531	5.076
	Cows	Clustering coefficient	0.011	5.9.10^−05^
		Average path length	7.184	7.516
**District level**	All Cattle	Clustering coefficient	0.57	0.50
		Average path length	1.79	1.49
	Cows	Clustering coefficient	0.39	0.25
		Average path length	2.03	1.74

The power law exponent α value oscillated around 2.5 for degree values >21 for the all cattle global network, and degree values >13 for the only cow global network. Practically, for each annual network, the α value was around 2.5, for all cattle (2014: 2.5; 2015: 2.4; 2016: 2.5; 2017: 2.4; 2018: 3.9) and the α value ranged from 2.5 to 2.6 for the only cow annual network. At the monthly level, the power law exponent α ranged from 2.5 to 3.0 for the degree values >21 **(S4 Fig)**.

#### District level

The distribution of node degrees in global networks showed a unimodal distribution **(Fig 8)**. The clustering coefficient and average path length of the real networks were similar to those of the random networks (**Table 5**), indicating that the global networks did not exhibit small-world properties.

### Percolation analysis

**Fig 9:**
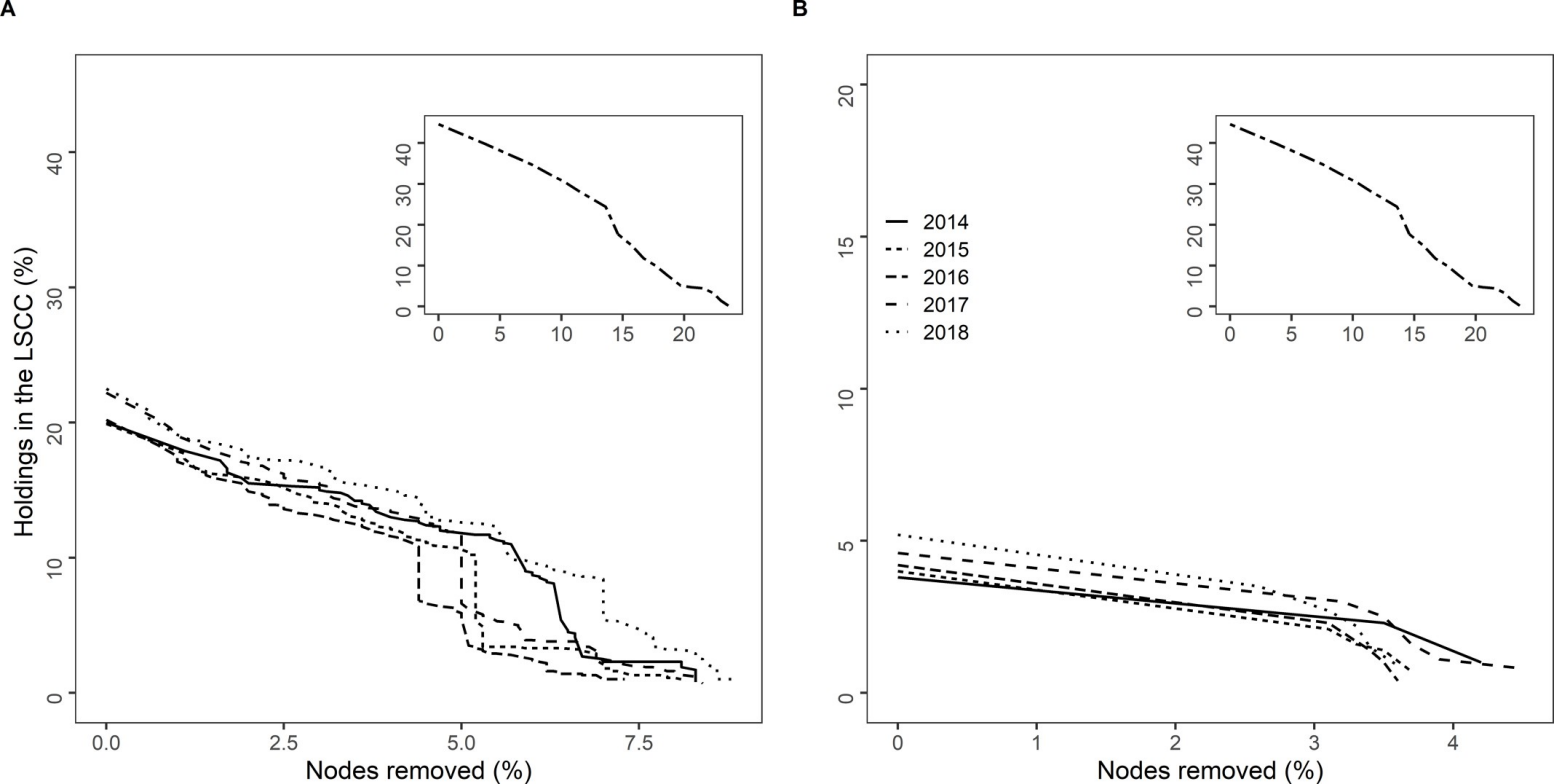
Percolation results for the global (inset) and annual networks (main graphs). (A): all cattle, (B): only cows in Paraguay.

No thresholds at which the LSCC would rapidly disappear were identified at the global level for either all the cattle network or the only cow networks **(Fig 9)**. At the annual level, from 4% to 6% of the nodes with higher betweenness should be removed to notice a fragmentation of the LSCC for the all cattle networks. For only cow networks, that threshold was around 3%. However, it should be taken into account that the size of the initial annual LSCCs represented only 20% of all nodes for all cattle networks and 4% for only cow networks.

## Discussion

Cattle movements in Paraguay between 2014 and 2018 were analyzed at two levels (holding and district) and at different temporal scales (monthly, annually, and globally), using the networks analysis methodology and network indicators. We also considered the implications for control and surveillance of two important diseases affecting cattle production through the use of: (i) all cattle data, as an approach to spread and control analysis in case of FMD re-introduction, and (ii) only cow data to describe relevant characteristics of the generated networks for surveillance and control of BB, an endemic disease, which mainly affects cows.

The fundamental characteristic of Paraguayan cattle raising is that it is extensive. Many cattle are raised in extensive farming systems in the western region (Chaco), which represents more than 60% of the country’s area. The Chaco region has a semi-arid climate, with pastures of high productivity, compared to the east which is a low fertile area with constant rains. The eastern regions have tropical and subtropical forests with a pleasant climate. These regions concentrate most of the human population as well as semi-intensive dairy farms. This regional contrast is reflected in the spatial density of farms found in this study, with a higher farm density in the east of the country.

The cattle trade data analyzed showed that a larger number of cattle movements occurred in the eastern region (2 to 3 times), which may be linked to its higher farm density compared to Chaco. Most movements of cows and cattle excluding cows occurred between farms, but more cows were moved to slaughterhouses than cattle excluding cows. This could be explained by the fact that young cattle included in the cattle excluding cow category are often moved to other farms for breeding or fattening.

The analyzed cattle movements included only 50% of the recorded cattle farms of the country. It is very likely that farms that did not recorded any movement are small properties that trade cattle with other small farms in their vicinity or do not exchange cattle at all. This phenomenon has been studied in other South American countries by estimating the number of unrecorded movements, such as reciprocal practices between neighbors or illegal movements [60, 61]. Surveys could be implemented on small cattle farms to determine their trade practices. Based on JI node values, which were not very high, holdings involved in all cattle/only cow movements were stable from one year or month to another. Between 50% and 60% of the global nodes were present in less than two years or in 4 or less months. It is very likely that those nodes correspond to small family farms that do not exchange cattle very often and that nodes that appear every year or more often at the monthly level are commercial farms or markets that commercialize cattle throughout the year. Similarly, JI link values were low, indicating that cattle trade did not occur systematically between the same holdings.

Holding networks in Paraguay had a stable heavy-tailed degree distribution over time, low average path lengths and an important variance-to-mean ratio of degrees. These characteristics would allow for a rapid and persistent spread in the case of a possible introduction of a disease like FMD or considering an endemic disease such as brucellosis, which could potentially affect most nodes if appropriate control measures are not taken [38, 62]. This is because many low degree nodes are connected to high degree nodes known as “hubs” that act as centers increasing the existing connections within the network. Disassortativity of the networks confirmed the presence of hubs. For animal movements, the main hubs are markets, like we found in our study, and they are considered holdings involved in super-spreading events. Because of their central position in the network, hubs are highly susceptible to infection and they play a pivotal role in the spread and maintenance of infection [63], so targeted surveillance and control methods applied in these holdings could provide effective benefits for disease control.

Many countries in South America have used network analysis as a veterinary epidemiological tool. For example, the analysis of static annual cattle networks in Uruguay made it possible to identify potential surveillance and control measures based on the heterogeneity of movement patterns and the identification of farms that could be involved in possible super-spreading events through indicators of centrality [64]. Likewise, in the case of cattle in Argentina, movements with high risk were identified for the months of April and June due to animal management characteristics. Districts with high degrees of connection were identified to plan possible future control measures [65]. In Brazil, suspension of vaccination against FMD was proposed in 2017 and therefore, several investigations were conducted, including a study on cattle movements. The results showed that connectivity and thus FMD transmission could be reduced with the elimination of nodes with high intermediation [60, 66].

The size of the LSCC has been considered an estimate of the lower bound of the maximum epidemic size in case of introduction of an exotic disease [50, 55]. The LSCC covered 44% of the holdings for the all cattle network at the global level, and had a wide geographic distribution across the country. This value is not far from that found for the holding network in Uruguay (51%) [64]. In contrast, at the district level, the LSCC for all cattle included all the districts in Paraguay, while in Argentina it included between 64% and 70% according to the season in the studied year [65]. At the monthly level, larger sizes of the LSCC of the all cattle networks were found between July and October, the period when young cattle are moved to fattening holdings. This could be a high risk period for FMD propagation.

Several studies have shown that performing node removal in a network with scale-free properties reduces the vulnerability of the network and limits the scope of a potential epidemic, as these networks tend to become unstructured upon node removal [39, 42]. We found that at the global level the LSCC would not be easily fragmented by removing the nodes with higher betweenness of the global networks at the holding level, which means that controlling the long-distance (i.e. trade-mediated) spread of FMD would imply the total stoppage of commercial cattle activities. This can be attributed to the small-world properties that the global networks exhibited at the holding level: even after the hubs were removed, the high clustering coefficient allowed the LSCC size to remain high. The studies conducted in other countries in South America have shown that cattle trade networks displayed scale-free properties for the whole country (Uruguay, Argentina) [65], or at a regional level (Mato Grosso do Sul in Brazil) [60]. An outbreak of an FMD serotype not covered by the vaccine currently implemented in Paraguay would imply large economic losses for the livestock sector. This concern is shared by neighboring countries that have also conducted network analyses of cattle movements. A shared characteristic of all these countries is that cattle raising is one of the main production sectors and that the risk of transborder spread of a highly transmissible disease, such as FMD, is not negligible, as has been shown in studies aimed at establishing FMD risk areas [66, 67].

The presence of properties similar to small-world networks and an important variance-to-mean ratio of degrees, at the holding level, for the global only cow network in Paraguay, suggests that trade could allow BB, like FMD, to spread far from an outbreak through markets, but also locally. In terms of control of BB and in a context of limited resources, community identification could allow the authorities to first target control measures in the less affected communities, in order to gradually constitute brucellosis-free subpopulations, which could later provide healthy animals to other communities. Communities at the district level could also facilitate the enforcement of control measures by zones in the country. Control measures could include a vaccination program and the requirement for BB vaccination certificates when trading cattle.

We used static networks that do not take into account the temporality of movements that could be fundamental to understanding disease dynamics. However, static networks are appropriate and widely used in veterinary epidemiology to understand network topology [68, 69], which was also the case in our study. Nevertheless, future studies on cattle movement data in Paraguay should integrate temporal network methods. For simplicity reasons we only considered non-weighted networks in the present study. Using the number of traded animals as link weights may change the results obtained as has been reported by some authors [70]. Concerning FMD, we have only considered cattle, but this disease also affects pigs and small ruminants. In addition, there are other transmission routes, more local than trade, between farms that raise susceptible species (airborne contagion, movement of people, vehicles and livestock equipment). These elements should be taken into account to understand the risk of FMD diffusion in Paraguay. However, as cattle is the main species raised in Paraguay, our study allowed us to analyze the main risk of FMD virus dissemination over long distances, induced by cattle trade.

In conclusion, the networks of cattle movements in Paraguay have properties similar to and small-world networks and an important variance-to-mean ratio of degrees that would favor the spread of animal infectious diseases in the country. The spread of an exotic FMD serotype would be difficult to control, and therefore effective surveillance measures should be implemented. Control of animal movements at the borders should be among the measures to enforce as part of a transborder FMD surveillance program with Brazil and Argentina, as has been suggested previously [66, 67]. Regarding BB control, scale-free and small-world properties should also be taken into account when designing control protocols.

## Supporting information

S1 Fig Monthly network indicators from 2014 to 2018 in Paraguay. Holding level (main graphs), district levels (insets), all cattle (plain lines) and only cows (dashed lines).
(TIFF)

S2 Fig Monthly Jaccard index values for nodes and links from 2014 to 2018 in Paraguay. Holding level (main graphs), district levels (insets), all cattle (plain lines) and only cows (dashed lines).
(TIFF)

S3 Fig Monthly largest strongly connected component size at the holding level from 2014 to 2018 in Paraguay. (A): All cattle, (B): Only cows, annual networks.
(TIFF)

S4 Fig Monthly power law exponent values at the holding level from 2014 to 2018 in Paraguay. All cattle (plain lines) and only cows (dashed lines).
(TIFF)
