# Supplementary figures and images for: Analysis of cattle movement networks in Paraguay: Implications for the spread and control of infectious diseases
Source: PLoS One. 2022 Dec 19;17(12):e0278999. doi: 10.1371/journal.pone.0278999 (PMC9762583; doi:10.1371/journal.pone.0278999)

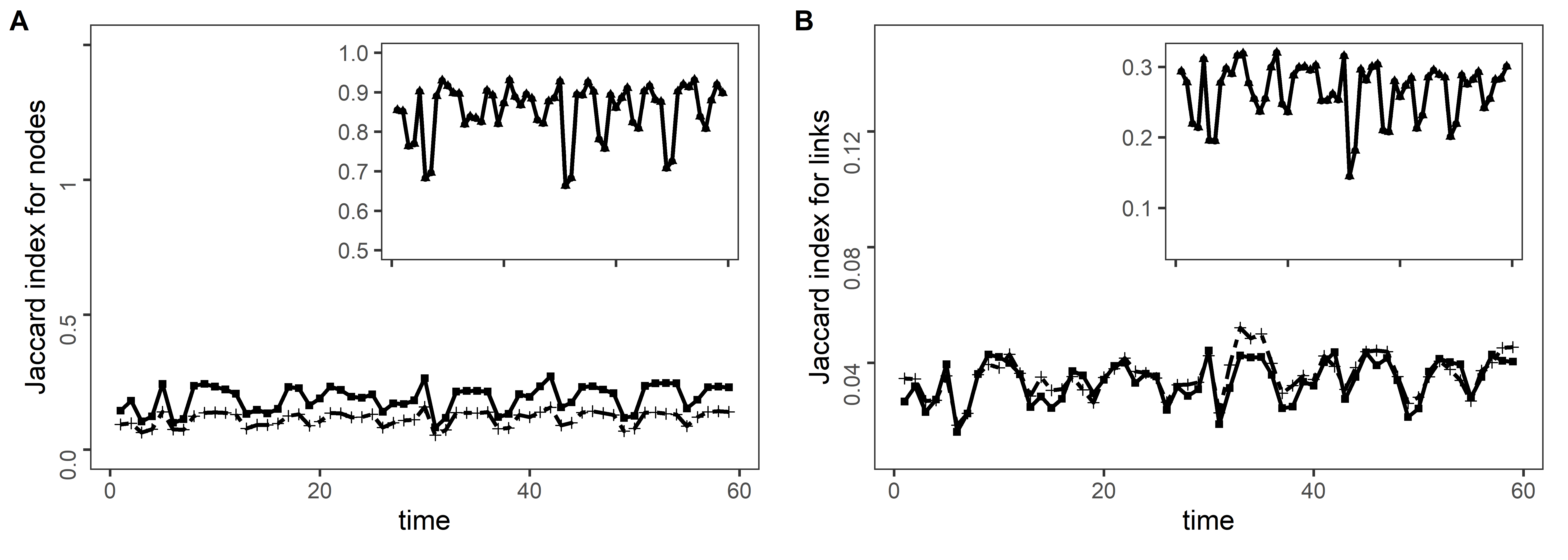

Supplement: S2 Fig — Holding level (main graphs), district levels (insets), all cattle (plain lines) and only cows (dashed lines). (TIFF) [file pone.0278999.s002.tiff]

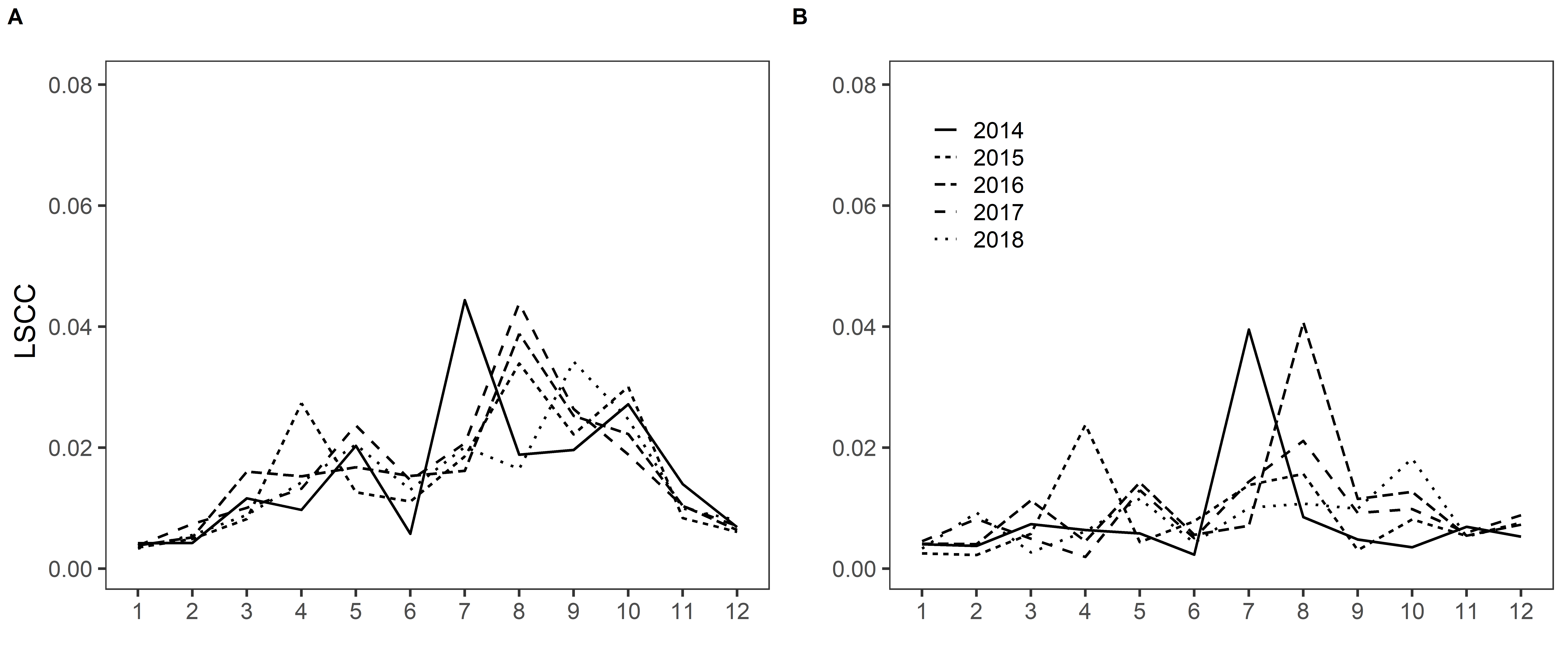

Supplement: S3 Fig — (A): All cattle, (B): Only cows, annual networks. (TIFF) [file pone.0278999.s003.tiff]

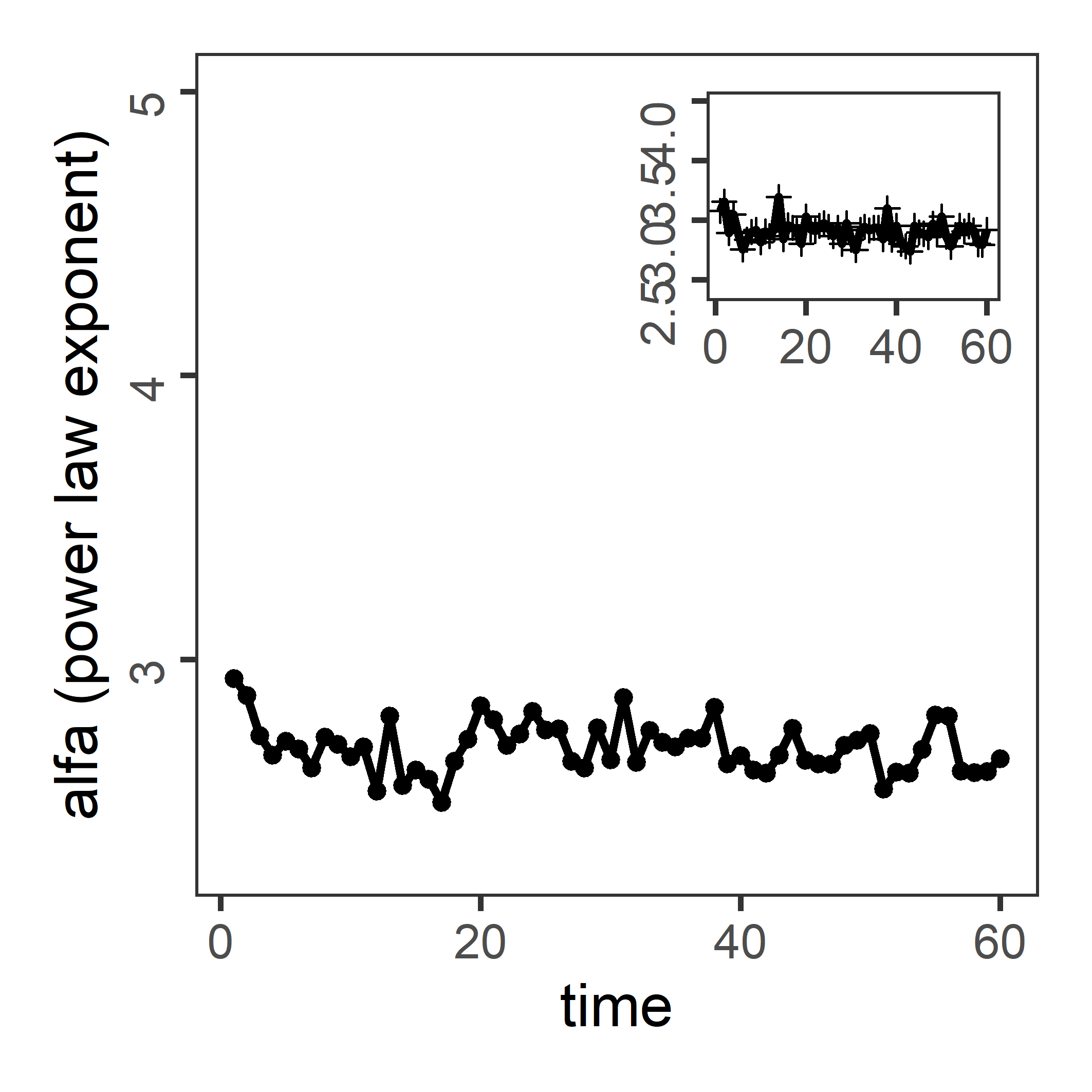

Supplement: S4 Fig — All cattle (plain lines) and only cows (dashed lines). (TIFF) [file pone.0278999.s004.tiff]
